# Functional imaging in living plants—cell biology meets physiology

**DOI:** 10.3389/fpls.2014.00740

**Published:** 2014-12-19

**Authors:** George R. Littlejohn, Tobias Meckel, Markus Schwarzländer, Alex Costa

**Affiliations:** ^1^Division of Plant and Microbial Sciences, School of Biosciences, University of ExeterExeter, UK; ^2^Membrane Dynamics, Department of Biology, Technische Universität DarmstadtDarmstadt, Germany; ^3^Chemical Signalling, Institute of Crop Science and Resource Conservation, University of BonnBonn, Germany; ^4^Department of Biosciences, University of MilanMilan, Italy; ^5^Milan Division, Institute of Biophysics, National Research CouncilMilan, Italy

**Keywords:** *in vivo* imaging, plants, organelles, cell physiology, dynamics, fluorescent protein sensors, quantitative microscopy

The last quarter of a century has brought major developments in the acquisition of images from plants through improvements in microscopy equipment, software and technique. Likewise, step changes in image analysis tools and the utilization and iterative redesign of fluorescent protein based markers and probes has revolutionized the ability of researchers to ask fundamental questions in cell biology and physiology. This research topic gives a snapshot of the current shape of the field in the plant sciences.

The articles contributed to this research topic are indicative of the work emerging from the plant imaging community and cover, variously, the characterization of individual protein functions; localization and interactions; the use of physiological biosensors; spectroscopic techniques, which utilize autofluorescence of plant tissues and label-free approaches; developmental studies and software engineering. These reflect the broad areas in which imaging is currently being used to functionally dissect plant processes.

A focus in this research topic is the quantitative imaging of fluorescent sensors to explore cell function.

Förster resonance energy transfer (FRET) and how sensitized emission may be used for quantification *in vivo* imaging is reviewed by Müller et al. (2013) who discuss a set of methods that allows for the analysis of molecular interactions, in the light of recent developments in fluorescence microscopy, which have achieved higher spatial and temporal resolution as well as a much-improved sensitivity. A comprehensive overview of FRET imaging is given with a focus on fluorescent proteins and the procedure and analysis of sensitized emission, which allows for a fast and repetitive monitoring of FRET efficiencies as required for the investigation of dynamic plant cells.

A perspective on the use of genetically encoded fluorescent biosensors (including FRET-based probes) in plants is given by Gjetting et al. (2013). The authors discuss the development of a rapidly growing repertoire of genetically encoded fluorescent sensors and how these developments have been a key driver for functional imaging over the last two decades as well as how new sensors have been adopted by plant biology and future opportunities. Autofluorescence from photosynthetic pigments and secondary metabolites, mounting techniques affecting physiological status, sensor silencing and plant specific compartments, such as the apoplast, are identified as technical burdens that can hamper straightforward sensor usage in plants. Plant-adjusted sensor design, such as the usage of new fluorescent protein variants, and imaging techniques, like fluorescent lifetime imaging (FLIM), are recognized as technical opportunities to advance *in vivo* sensing in plants. Biological promise comes from bespoke sensing approaches in which the sensor is matched current questions of plant metabolism, physiology and signaling, such as sugar homeostasis, hormone regulation and pH dynamics of acidic compartments.

The development and properties of pH probes as one group of the genetically encoded sensors discussed by Gjetting et al. (2013) is given detailed attention and set in biological context by Martinière et al. (2013). Imaging of intracellular pH dates back to the early efforts to exploit fluorescent proteins as sensors for *in vivo* physiology. A still growing repertoire of sensor variants for pH has been extensively applied in plant cells to understand subcellular pH milieus and proton gradients across membranes. Nevertheless pH dynamics on a cellular scale are far from understood and potential functional roles of pH as a central physiological parameter are often elusive. In their perspective article, Martinere et al. shine a spotlight on the opportunities and the persisting technical constraints of pH imaging in plants. Insights gained from *in vivo* pH imaging are discussed with respect to exocytotic function, root apoplast responses to changing environments and growth. The highly dynamic nature of the archipelago of subcellular pH islands is exemplified for the physiological transition within the endomembrane system between endoplasmic reticulum (ER) and vacuole.

These three review articles set the scene for two original research articles, contributed by Aller et al. (2013) and Xiong et al. (2014) who report the development of new tools for *in vivo* imaging of glutathione redox status and Ca^2+^, respectively. Aller et al. (2013) introduce a new member of the redox sensitive GFP family, named roGFP2-iL. roGFP sensors have been extensively used for the *in vivo* monitoring of glutathione redox potential in both animal and plant cells. The founding members of the family, roGFP1 and roGFP2, have midpoint potentials of −290 and −280 mV respectively, compatible with the monitoring glutathione redox status in plasmatic compartments such as cytosol, mitochondria and peroxisomes. The newly developed roGFP2-iL, which shows a midpoint potential of −238 mV, now enables measurement of glutathione redox status under more oxidizing circumstances, such as in genetic backgrounds with impaired thiol redox maintenance (here exemplified for the glutathione deficient *Arabidopsis rml1* mutant) or in the ER, where both roGFP1 and roGFP2 are completely oxidized. This makes a powerful enhancement of the toolset of glutathione redox sensors and shifts the redox frontier allowing to explore new biology not only in plants.

Addressing another hub of regulation, Xiong et al. (2014) introduce a Bioluminescence Resonance Energy Transfer (BRET) sensor for Ca^2+^ in *Arabidopsis*. The BRET sensor “re-unites” old partners from the jellyfish *Aequoria victoria*—aequorin and GFP, enabling imaging of Ca^2+^ dynamics in entire seedlings and mature leaves of *Arabidopsis* without the necessity of illumination, as required for other popular Ca^2+^ sensor classes, such as the Yellow Cameleons, the GCaMPs and Case12. Instead the GFP-aequorin (G5A) sensor harnesses the photons emitted by the aequorin-coelenterazine complex upon binding of Ca^2+^ to excite the adjacent GFP, via BRET, the fluorescence of which can be detected with a cooled charge-coupled device camera (CCD). This approach allows for increased sensitivity as compared to standard aequorin-based Ca^2+^ sensing and enabled the authors to monitor long-distance Ca^2+^ waves propagating throughout the entire plant body after salt stress treatment applied to the root. This new Ca^2+^ imaging approach will complement other recently developed tools for the *in vivo* analysis of this central second messenger.

Instead of applying fluorescent dyes or introducing recombinant sensor proteins, the same autofluorescence by endogenous plant compounds highlighted as a burden for quantitative imaging by Gjetting et al. (2013) may be actively exploited to provide valuable physiological insight. New spectroscopic techniques for label-free imaging to investigate plant physiology are presented by Peter et al. (2014) and Conejero et al. (2014).

Peter et al. (2014) use spectro-microscopy and Statistical Analysis of Room Temperature Emission Spectra (SART) to characterize *in vivo* function of photosystems PSI and PSII in chloroplasts. This non-invasive technique exploits the natural light absorbance properties of chloroplasts and has the ability to deliver photosynthetic parameters for single chloroplasts at normal growth conditions. As this technique requires only moderate modification of a confocal microscope, it may be readily implemented by well-resourced laboratories.

Conejero et al. (2014) present a method combining spectral analysis with linear unmixing to facilitate histolocalization of phenolics in coffee leaves. Their protocol involves two-photon excitation, spectral characterization of pure chemicals and advanced linear unmixing. Conejero et al. (2014) are able to follow the amount and distribution of key phenolic compounds throughout the development of leaves of various *Coffea* species. By obviating the need for any staining, truly non-invasive histochemical analysis based on quantitative imaging is achieved.

Label-free SRS microscopy is used by Littlejohn et al. (2014) to delimit the negative-space in plant leaves in their paper updating the use of perfluorocarbon mounting media in plants leaves. Functional imaging in intact, living leaves as the main organ of photosynthesis, is often particularly desirable. Much work has been performed on leaf epidermis, while high quality imaging of the mesophyll or vascular bundle cells can prove challenging, due to the optical complexity of the tissue with multiple stacked cell layers and air spaces. Littlejohn et al. (2014) empirically assess the usage of perfluorocarbons, as non-aqueous and non-toxic mounting media for *in vivo* microscopy. A systematic comparison of yet untested perfluorocarbons with four state-of-the-art microscopy techniques pinpoints strong advantages for image quality from the use of perfluoroperhydrophenanthrene. This methodological advance goes far beyond producing “prettier images” and opens a new window for the quantitative *in vivo* study of a defining plant tissue. A particular benefit may be anticipated for ratiometric sensing approaches of physiology where increasing noise and chromatic aberrations in deeper tissue layers can hamper accurate quantitation.

Sattarzadeh et al. (2013) provide an example of how the use of confocal and spinning disc microscopy and *in vivo* imaging facilitates the definition of subcellular localization of proteins through the generation of chimeric fusion constructs between a fluorescent protein (e.g. GFP, YFP, RFP) and the full protein of interest or a functional domain. The authors identify conserved 42 amino-acid PAL domains present in 12 of the 13 *Arabidopsis* class XI myosin isoforms. YFP translational fusions for 11 different myosin PAL sub-domains allowed determination of their subcellular localization at Golgi, mitochondria, nuclear envelope, the plasma membrane and unidentified vesicles. Interestingly, the simultaneous expression of three PAL sub-domains resulted minimal or negligible movement of Golgi and mitochondria, allowing the authors not only to demonstrate that different YFP-PAL sub-domains localize to different subcellular compartments, but also that their overexpression can interfere with the mobility of the marked compartments in the cell. This work illustrates the elegance of *in vivo* imaging in exploring dynamic cell biological processes.

To extract the relevant information out of the highly complex dataset that is an image quantitative evaluation is essential, but far from trivial. In the field of plant cell morphology, cell shapes are irregular and highly variable, which requires the use of quantitative techniques to accurately define shape and provide well-defined phenotypic descriptions. In their review Ivakov and Persson (2013) present the current state of knowledge on cell shape formation in plants, focusing on the use of new quantitative tools and algorithms required to quantify and compare cell shapes in 2D and 3D obtained from microscope images.

This research topic reflects the breadth of approaches developed, adjusted and followed by the plant community in terms of sample preparation and image acquisition and analysis. A major driver behind the recent progress on the burning questions in plants sciences have been technological advances in imaging. Yet the field is far from maturity and progresses quickly with the promise of keeping delivering fundamental new insights in the years to come.

## Conflict of interest statement

The authors declare that the research was conducted in the absence of any commercial or financial relationships that could be construed as a potential conflict of interest.
